# A Global Seawater Density Distribution Model Using a Convolutional Neural Network

**DOI:** 10.3390/s24061972

**Published:** 2024-03-20

**Authors:** Qin Liu, Liyan Li, Yan Zhou, Shiwen Zhang, Yuliang Liu, Xinwei Wang

**Affiliations:** 1 Optoelectronic System Laboratory, Institute of Semiconductors, Chinese Academy of Sciences, Beijing 100083, China; liuqin@semi.ac.cn (Q.L.); zhouyan@semi.ac.cn (Y.Z.); shiwenzhang@semi.ac.cn (S.Z.); ylliu@semi.ac.cn (Y.L.); wangxinwei@semi.ac.cn (X.W.); 2 Center of Materials Science and Optoelectronics Engineering, University of Chinese Academy of Sciences, Beijing 100049, China

**Keywords:** seawater density, spatial distribution model of density, latitude, convolutional neural network

## Abstract

Seawater density is an important physical property in oceanography that affects the accuracy of calculations such as gravity fields and tidal potentials and the calibration of acoustic and optical oceanographic sensors. In related studies, constant density values are frequently used, which can introduce significant errors. Therefore, this study employs a basic convolutional neural network model to construct a comprehensive model showing the seawater density distribution across the globe. The model takes into account depth, latitude, longitude, and month as inputs. Numerous real seawater datasets were used to train the model, and it has been shown that the model has an absolute mean error and root mean square error of less than 1 kg/m^3^ in 99% of the test set samples. The model effectively demonstrates the influence of input parameters on the distribution of seawater density. In this paper, we present a newly developed global model for distributing seawater density which is both comprehensive and accurate, surpassing previous models. The utilization of the model presented in this paper for estimating seawater density can minimize errors in theoretical ocean models and serve as a foundation for designing and analyzing ocean exploration systems.

## 1. Introduction

Seawater density is a fundamental physical property in the field of oceanography. The study of seawater density distribution, variability, and influencing factors can aid in understanding scientific issues such as the dynamic properties of oceans, ecosystems, and climate change. Using a spatial distribution model for seawater density in the region of interest would significantly decrease errors in gravitational field modeling by using actual density distribution [1]. The effect of seawater density variations on tidal potential is as large as 2–3 cm in water height equivalent [2]. In comparison to homogeneous seawater, the total speed of a tsunami in density-stratified seawater is lower [3]. The relationship between the density of seawater and the rise in sea level is amplified within the framework of global warming [4]. Furthermore, seawater density acts as a vital point of reference or compensation value for the calibration and adjustment of ocean sensors [5]. For example, the density of the medium needs to be considered in the sound velocity variation in sonar detection technology [5] and high-precision optical ocean detection technology [6]. Previous studies have generally regarded seawater density as unvarying, but this notion is inadequate [7]. The density of seawater varies between 995–1070 kg/m^3^. If a constant density is used in a study, errors will inevitably occur. For instance, using constant density in gravity calculation models can introduce an error of up to 2% [8]. Understanding seawater density distribution can be advantageous for technological advancements in marine applied sciences.

Measuring seawater density directly in situ poses challenges [9]. Indirect measurement techniques include remote observation using satellites and radar to obtain sea surface information for inferring sea surface density, which has wide coverage but can only measure the sea surface. In situ observation using buoys and submersibles is also used to measure temperature, salinity, pressure, and other parameters in the ocean to calculate seawater density, which has high accuracy but is easily affected by the marine environment. For researchers, seawater temperature, salinity, and depth information can be obtained from marine hydrographic datasets. These datasets can then be used to calculate seawater density with the thermodynamic Equation of Seawater-2010 (TEOS-10) [10]. Accessing these datasets is easy, and shared resources provide valuable sources for marine-related research, such as the World Ocean Database [11], the Copernicus Marine Environment Monitoring Service, and the Global Ocean Forecast System. The accuracy of TEOS-10 calculations is exceptionally high [12]. However, density in this method depends on temperature, salinity, and pressure rather than latitude and longitude. A highly accurate dataset of seawater density distributions can be formed using densities calculated by TEOS-10, along with spatial and temporal data from marine datasets. However, many of these data calculations and analyses are redundant for the aforementioned applications. Building a generic model from this dataset of seawater density distribution is desirable to increase efficiency and reduce computational costs.

Research into the global distribution of seawater density is limited. Neutral density γ^n^ and neutral surfaces [13] provide an appropriate framework for ocean model calculations and analysis. The sampling points of latitude, longitude, and depth have high resolution. Gladkikh and Tenzer [14] developed a functional model that provides an overall understanding of seawater density distribution based on latitude and depth. Their model employs the absolute latitude yet does not consider the dissimilarities between the Northern and Southern Hemispheres. Furthermore, Talley’s investigation demonstrated that seawater density varies according to latitude and depth, across seasons, and among oceans [15]. The density of seawater undergoes significant variation with changes in depth and latitude. The researchers also accounted for changes in longitude [16] and month [17]. Modeling the effects of these variables set in advance is challenging.

In recent years, oceanographic researchers have employed deep learning to develop seawater temperature, salinity, and tide prediction models [18,19,20], achieving some success. This in turn provides a sound basis for establishing seawater density within the scope of this paper. Compared with several classical deep learning methods, the convolutional neural network (CNN) is an optional base used to build a density model. CNNs are deep learning models that have shown exceptional proficiency in solving complex nonlinear issues across various industries [21].

This current study strives to fabricate a seawater density distribution model with a CNN to introduce the impact of different seasons and ocean regions, thereby creating a more authentic model. The study scrutinizes the model’s accuracy as well as its ability to reflect alterations in each given factor. It is hoped that a convenient and accurate mathematical tool can be provided for theoretical studies and detection techniques affected by the density distribution of seawater.

## 2. Materials and Methods

### 2.1. Dataset

Data from the Argo program, which forms part of the Global Ocean Observing System, were used to collect oceanic information such as temperature, salinity, pressure, and biogeochemical components [22]. The International Argo Programme and its associated national programs offer these data freely. Most importantly, the Argo dataset was selected for research requirements due to the following reasons [23]. (1) The vertical sampling resolution is “hybrid”. Where the vertical profile varies greatly, the resolution is high but not less than 1 dbar. Conversely, where the vertical profile varies little, the resolution is low, up to 50 dbar. (2) The majority of the floats operate within a pressure range of up to 2000 dbar from 2016. (3) The geographic distribution of samples offers almost complete ocean coverage. The Argo project covers a vast expanse of the Atlantic, Pacific, and Indian oceans, with only a few floats deployed on continental margins and in the Arctic Ocean. (4) The temperature, salinity, and pressure sensors have high accuracy and exhibit good stability. The accuracy is 0.002 °C for temperature, 2.4 dbar for pressure, and 0.01 PSS-78 for salinity, after delayed-mode adjustments.

Data collected from 2017 to 2022 were analyzed, and ‘data quality code’ options were configured to ensure reliability. The necessary parameters of the dataset are DATE, LATITUDE (degree_north), LONGITUDE (degree_east), PRES (decibar), PSAL (psu), and TEMP (degree_Celsius). Every parameter marked with ‘XX_QC = 1’ indicates good quality control.

The dataset was computed and approximated. Given that Argo’s floats have a measurement period of 10 days, the data’s temporal resolution can be set to one month. Therefore, seasonal information was represented by extracting the months from ‘DATE’. In-situ density was computed using TEOS-10, after converting practical salinity to absolute salinity and in situ temperature to conservative temperature. Subsequently, the latitude and longitude were rounded to a resolution of 1°. Increasing the resolution scale of latitude and longitude aims to enhance the amount of data on each location cell when the data is non-uniformly distributed in terms of location. This facilitates the convergence of the model later. Note that ‘PRES’ has not been converted to depth. For consistency, we will use ‘depth’ instead of ‘PRES’. We have excluded a minor portion of the data due to objective factors, such as locations not considered part of the ocean in salinity calculations. The data beyond 2200 dbar were removed because they were too insignificant for use in the CNN.

The dataset needed to be divided into training, validation, and test sets for input into the neural network. Their respective tasks are debugging parameters, model optimization, and generalization evaluation [24]. The ratio should be approximately 6:2:2. The data from 2017 to 2021 were randomly distributed into training and validation sets at an 8:2 ratio. To ensure the test set has an extensive range of data distributions, we designated the data for the entirety of 2022 as the validation set. Centering and scaling were performed on each variant independently by computing the mean and standard deviation of the samples in the training set. This process is known as standardization. The mean and standard deviation were then stored and utilized for the validation and test sets. This method facilitated the creation of the necessary database.

### 2.2. CNN Architecture

**Figure 1:**
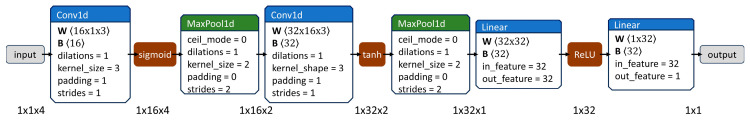
An illustration of the architecture of the CNN.

The network’s architecture is outlined in Figure 1. The model’s design incorporates a basic structure consisting of two 1D convolutional layers (with two Max pooling layers) and two fully connected layers. The settings, inputs, and outputs for each layer are shown in Figure 1.

Convolutional layers, activation functions, and pooling layers are standard tertiary structures in convolutional networks. The convolutional layer’s objective is to extract input data features by performing the convolution operation, reduce network complexity through parameter sharing and local perception to prevent overfitting, and enhance model generalization capability. Rectification involves the application of an activation function to the output of the convolutional layer. The pooling layer primarily simplifies network complexity. The fully connected layer changes the two-dimensional features produced by the convolutional layer into one-dimensional vectors. The book [25] provides a detailed description of each layer’s role.

We utilized the fundamental CNN architecture and purposely selected the activation function. The density exhibits an exponential relationship with depth [14,15]. Consequently, we utilized corresponding activation functions following the convolutional layers, namely sigmoid and tanh. Fully connected layers benefit from the nonlinear properties of ReLU.

Sigmoid is defined as:

Sigmoid(x) = 1/(1 + exp(−x)),(1)

Tanh is defined as:

Tanh(x) = (exp(x) − exp(−x))/(exp(x) + exp(−x)),(2)

ReLU is defined as:

ReLU(x) = max(0,x),(3)

**Figure 2:**
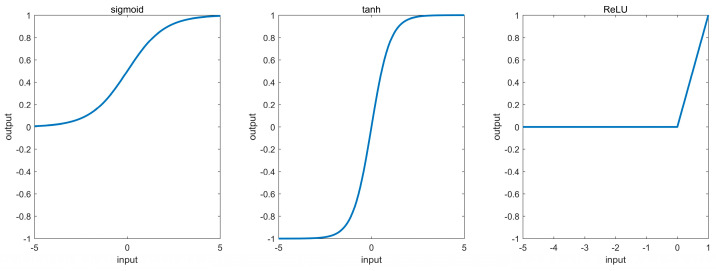
Activation functions used in the model.

The characteristic curves of the activation functions used are shown in Figure 2.

**Figure 3:**
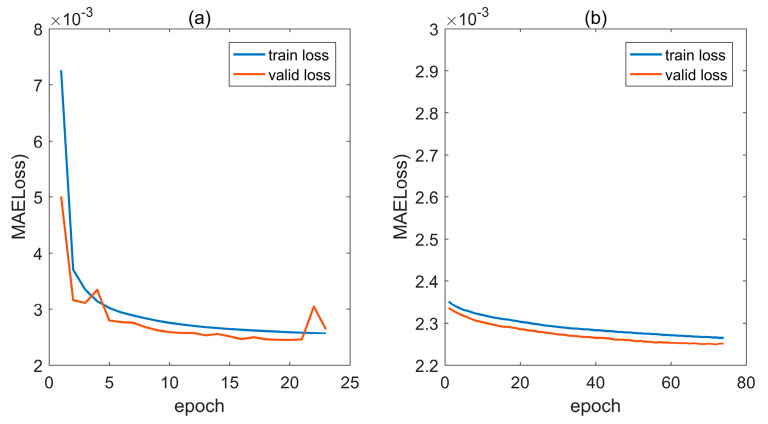
Model training convergence curves. The learning rate is 0.001 in (**a**) and 0.0002 in (**b**).

PyTorch was used to implement the model architecture depicted in Figure 1. The model learning setup employed MSELoss as the loss function and RMSEprop as the optimizer. The learning rate was set to 0.001 for the first training period and 0.0002 for the second. The convergence curve is shown in Figure 3. If the valid loss did not decrease for three consecutive epochs, training was stopped. Finally, the 68th model with the lowest validation loss was selected as the seawater density estimation model.

## 3. Results

The dataset from 2017 to 2021 serves as both the training and validation sets for the model demonstrated in Figure 1. The model’s performance is optimized by adjusting its hyperparameters. Consequently, a CNN model is developed to portray the in situ seawater density to latitude, longitude, depth, and month. The types and ranges of the input variables are presented in Table 1. The density of seawater in situ is provided by the model output, with data accuracy determined by statistical errors. This study analyzes the model’s output error and investigates the impact of each input variable on the output.

The 2022 dataset was utilized to test the model’s seawater density estimation capabilities. Using the TEOS-10 equation, the density was calculated as a reference value. The model outputted an estimated density which was then compared to the reference value, and the resulting error was subjected to statistical analysis.

**Figure 4:**
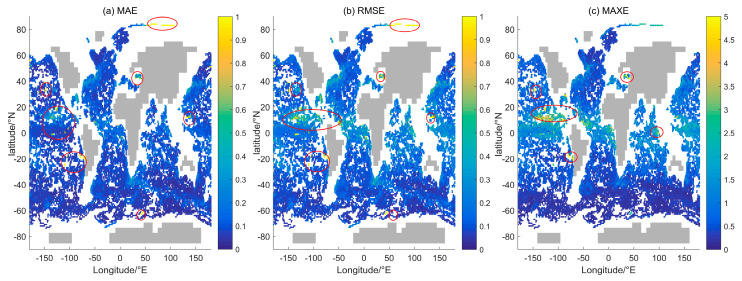
Error distribution of the test set. (**a**) MAE; (**b**) RMSE; (**c**) MAXE. The gray area represents the land and is determined by the output *in_ocean* = 0 of *gsw_SA_from_SP* in TEOS-10.

The test set’s latitude and longitude positions were derived using the *gsw_SA_from_SP* function from the TEOS-10 equation, setting *in_ocean* = 1. This criterion ensures that the sample data are not on land but may encompass inland seas. The error distribution for all of the latitude and longitude locations in the test set is shown in Figure 4, comprising absolute mean error (MAE), root mean square error (RMSE), and maximum absolute error (MAXE). The locations with MAE ≥ 1 kg/m^3^ are marked with red circles in Figure 4a, which account for 0.44% of the sample size of the test set, including the Black Sea, the East Pacific Ocean, and the Arctic Ocean. The Black Sea exhibits a unique two-layer density structure [26], resulting in a relatively large estimation error for a generic model. In contrast, the eastern Pacific Ocean is affected by the Peru and Californian currents, which are well-known cold currents with low temperatures and salinity that flow towards lower latitudes [15]. Sea ice significantly affects the waters of the Arctic Ocean. The dataset only includes a small proportion of samples from areas above 75° N (0.4%), which may contribute to the large errors in the density estimates of the Arctic Ocean. Figure 4b demonstrates that the root mean square error (RMSE) distribution is similar to the MAE distribution in Figure 4a. Locations where the RMSE is at least 1 kg/m^3^, constituting 0.72% of the test set sample, are similarly marked in Figure 4a by red circles. Figure 4c illustrates the distribution of MAXE. The red circles pinpoint the locales with MAXE ≥ 5 kg/m^3^, constituting 0.54% of the test set sample. Overall, the study proposes a highly accurate model for density calculation in most oceanic regions.

**Figure 5:**
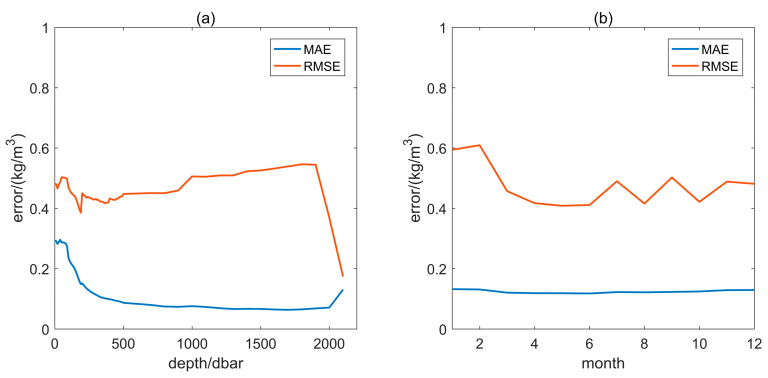
Error curves of the test set. (**a**) Error of depth and (**b**) error of month.

Figure 5 shows the error distribution curves for two other inputs to the model—depth and month. In Figure 5a, the MAE and RMSE for the density corresponding to depth are less than 0.3 and 0.546 kg/m^3^. The larger MAE is mainly in the range of 0–300 dbar. The large change in error level at depths greater than 2000 bdar is the result of a sudden drop in sample size. In Figure 5b, the MAE and RMSE of the month corresponding to the density are less than 0.132 and 0.609 kg/m^3^. The difference in error between months remained relatively stable.

## 4. Discussion

Previous seawater density distribution models exclusively utilize latitude and depth as independent variables in the density function. This research paper expands on this by incorporating two additional independent variables, longitude and month. Upon conducting a correlation analysis of the dataset, Pearson’s correlation coefficients of all seawater densities to depth, latitude, longitude, and month were found to be 0.97, −0.022, 0.019, and −0.014, respectively. The correlation coefficients for densities with inputs at depths less than 200 dbar are 0.48, −0.26, 0.05, and −0.077, respectively. The influence of longitude and month must be factored in for both shallow and deep waters. The significance of accounting for longitude and month in both shallow and deep waters is evident. The function of each independent variable represented in the model is evaluated individually below. Please note that the following analysis does not cover regions above 80° latitude due to the fact that sea ice density must be calculated separately in TEOS-10 [10], as well as the extreme errors at high latitudes shown in Figure 4.

**Figure 6:**
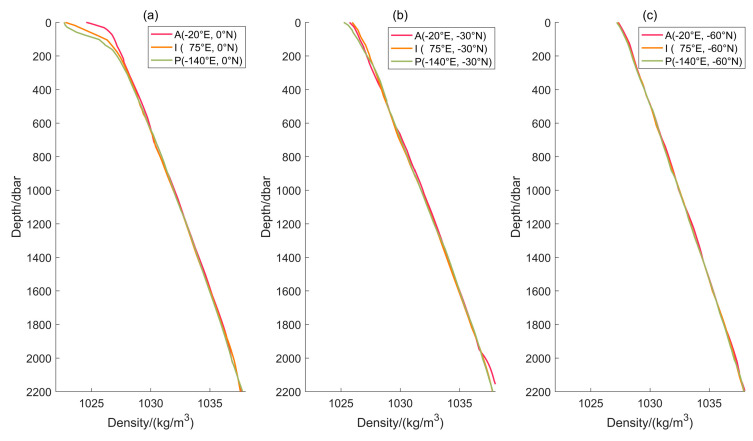
The model’s seawater density output varies with depth. There are density profiles (**a**) at 0° N, (**b**) at −30° N and (**c**) at −60° N respectively. The legend clarifies that the letters ‘A’, ‘I’, and ‘P’ correspond to the Atlantic, Indian, and Pacific oceans, respectively. The month of June is considered winter in the southern hemisphere.

Depth is the variable that correlates most highly with seawater density among the four independent variables. Figure 6 illustrates the correlation between seawater density and depth, which was calculated using the density model in this study. The curve of seawater density with depth is similar to the mathematical model proposed by Gladkikh and Tenzer [14]. The location selected for this analysis is situated far from the land. When comparing Figure 6a–c, the density increases as latitude increases at the sea surface, but the increment decreases with depth. Each subplot features density profiles from the Atlantic, Indian, and Pacific oceans. It is noticeable that the Atlantic Ocean displays a slightly greater density than the other two oceans in the same latitude near the sea surface. Figure 6a shows that the Atlantic Ocean is approximately 2.05 kg/m^3^ denser than the Indian Ocean and about 3.05 kg/m^3^ denser than the Pacific Ocean at a depth of 33 dbar. Large river outflows (Amazon, Congo, and Orinoco) cause the lowest surface densities in the Pacific Ocean in the tropics [15].

What is more, it is reasonable to expect that the fold lines may not be perfectly smooth. The variations in the density profile appear wavy, particularly in shallow regions. The CNN model is able to learn and account for these variations, which may not be captured by the mathematical expression for the density model.

**Figure 7:**
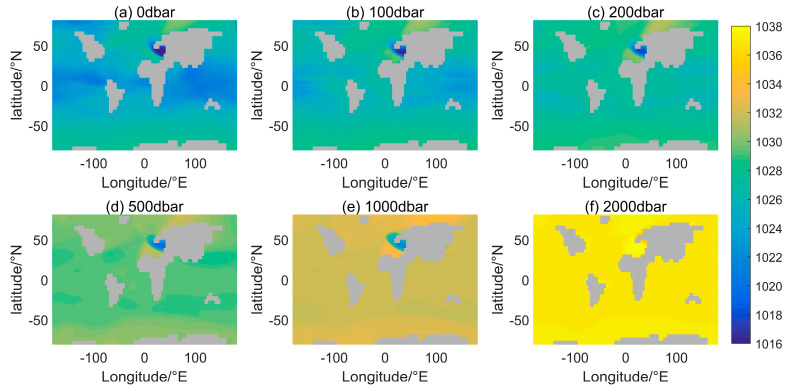
Annual mean sea water density distribution at different depths. “Annual” refers to the average of the results of the model calculations from January to December.

Latitude and longitude are both independent variables and their effect on the distribution of seawater density will be analyzed together in Figure 7. The annual average density is calculated without considering the month’s effect. Figure 7a displays the density distribution at a depth of 0 dbar, which represents the seawater surface density. The seawater surface density increases with latitude in the range of 1020–1030 kg/m^3^ when observing latitudinal change. This result aligns with Gladkikh and Tenzer’s model [14]. Furthermore, the distribution outcomes in Figure 7a are akin to those in Talley’s Figure 4.19 [15]. Nevertheless, in the zone of Europe where they meet the Arctic Ocean (0–40° E, 50° N), the aforementioned density drops beneath 1020 kg/m^3^. Significant areas of low density can also be found along the eastern coast of Russia near the Arctic Ocean. However, this area is where the error is large, as shown in Figure 4. Therefore, Figure 7a does not accurately depict this information.

Changes in the distribution of seawater density, in terms of the direction of change in longitude, occur mainly at continental margins. Ocean currents change the direction of motion in these regions, allowing the mixing of seawater, with temperature and salinity differences at different latitudes and depths. It seems from Figure 7d that seawater density varies with longitudinal distribution up to a depth of 500 dbar. Another manifestation of the variation in density with longitudinal distribution is the difference in density of different oceans separated by land. The main manifestations are in the North Atlantic and the North Pacific. The North Atlantic is affected by cold water from the high latitude regions of the Arctic Ocean [15] and is at least 1 kg denser than the North Pacific at the sea surface. And the coldest waters of the three oceans in the Southern Hemisphere are near Antarctica. Thus, the density of the seawater in the southern hemisphere is more homogeneous in the latitudinal direction than in the northern hemisphere.

**Figure 8:**
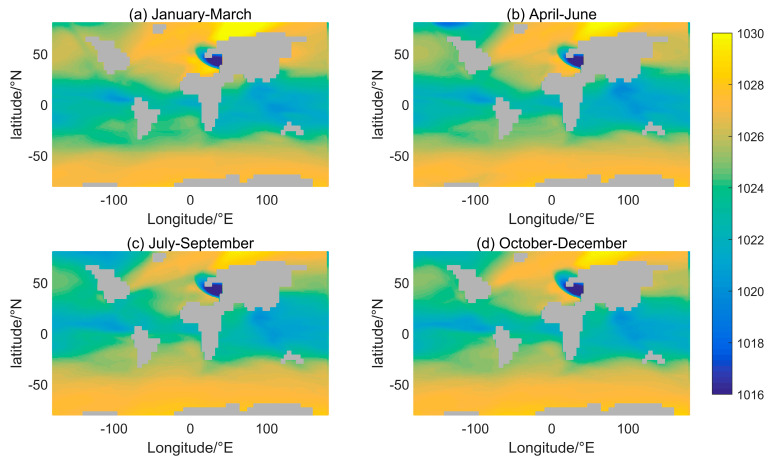
Monthly mean surface density distribution in different seasons.

Seasonal changes in solar thermal radiation impact the density distribution of seawater, which typically affects shallow seawater. To examine the seawater density distribution over time, Figure 8 illustrates the mean surface density for three months, corresponding with the seasons. In addition, the direct sunlight point fluctuates between ±23.5° N. The density variations represented in Figure 8 are primarily concentrated in the mid- and low-latitude regions. As shown in Figure 8b, high temperatures can be observed in the low-latitude region of the Northern Hemisphere, and to the north of the equator, a blue–green low-density band is distributed. Moving to Figure 8c, the blue–green low-density band expands in size and spreads to both sides of the equator. In Figure 8d, the blue–green low-density band is primarily located in the low-latitude region of the Southern Hemisphere. Both Figure 8a,c depict intermediate transition results between Figure 8b,d. In the mid-latitude region of the Northern Hemisphere, the sea surface densities exhibit substantial seasonal characteristics in the North Atlantic and North Pacific.

## 5. Conclusions

This paper presents a CNN model for modeling the global density distribution of seawater, eschewing explicit mathematical functions. The model inputs variables such as depth and latitude, traditionally used by mathematical functions, along with new variables like longitude and month. The training dataset for the model is sourced from Argo ocean data spanning 2017–2021. In contrast, the test set for error analysis uses Argo ocean data solely from 2022. Density values were determined using the TEOS-10 equation. The dataset was limited to a depth range of 2200 dbar.

The precision of the seawater density distribution model formulated in this research was enhanced by augmenting the input parameters. The values of MAE and RMSE for 99% of the input ocean regions do not surpass 1 kg/m^3^. The analysis of model outputs’ density distribution indicates that the model properly represents the correlation between seawater density and depth, latitude, longitude, and month. The seawater density distribution estimated by the model in this paper is substituted for the constant density and is used to analyze the impact of seawater density variations in related studies. This may be beneficial for theoretical analysis to reduce error and improve the accuracy of the detection technique.

## Figures and Tables

**Table 1:** Input specifications for the seawater density model.

Input	Resolution	Range
Latitude	1°	−90–90° N
Longitude	1°	−180–180° E
Depth	1 dbar	0–2200 dbar
Month	1	1–12

## Data Availability

Data are contained within the article and Supplementary Materials.

## Supplementary Materials

The Argo seawater data used in the paper can be downloaded at: https://dataselection.euro-argo.eu/ (accessed on 27 February 2024). The software for TEOS-10 is available from www.TEOS-10.org (accessed on 29 November 2021). The data and code for the model in this paper are accessible at: https://github.com/lqaaaaaaa/lq-seawater-density.git (accessed on 14 March 2024).
